# Electrophilic Selenium Catalysis with Electrophilic N-F Reagents as the Oxidants

**DOI:** 10.3390/molecules22050835

**Published:** 2017-05-19

**Authors:** Ruizhi Guo, Lihao Liao, Xiaodan Zhao

**Affiliations:** Institute of Organic Chemistry & MOE Key Laboratory of Bioinorganic and Synthetic Chemistry, School of Chemistry, Sun Yat-Sen University, Guangzhou 510275, China; grz616263@163.com (R.G.); liaolih@mail2.sysu.edu.cn (L.L.)

**Keywords:** alkenes, electrophilic fluorinating reagents, selenium catalysis, regioselectivity, stereoselectivity

## Abstract

A suitable oxidative system is crucial to electrophilic selenium catalysis (ESC). This short review offers the overview of recent development in ESC with electrophilic N-F reagents as the oxidants. Several highly selective transformations of alkenes such as allylic or vinylic imidation, pyridination, *syn*-dichlorination, oxidative cyclization and asymmetric cyclization have been described.

## 1. Introduction

Functionalization of alkenes is a perpetual goal in organic synthesis. One of the attractive routes to elaborate the carbon–carbon double bond of alkenes is through electrophilic selenium reagent-promoted selenofunctionalization. In this context, several electrophilic organoselenium reagents ArSeX (X = Cl, Br, OTf, etc.) have been developed and widely applied in routine synthesis [1,2,3,4,5,6,7,8,9]. In general, the introduced selenium moiety was further modified via oxidative or reductive manner leading to the formation of non-selenium-containing products. This process was not green and wasted stoichiometric selenium reagents. From the view of atom economy, accomplishing the transformation with a catalytic amount of organoselenium compounds is environment-friendly and highly desirable.

**Scheme 1:**
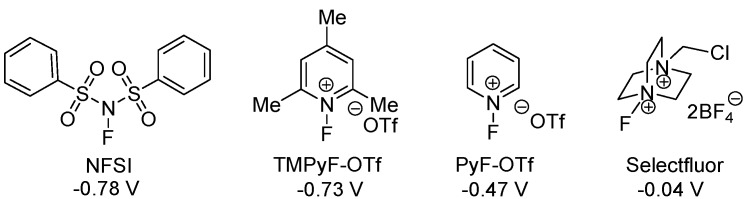
Electrophilic N-F reagents applied in ESC process (redox potentials vs. SCE).

A selenenylation–deselenenylation process, namely electrophilic selenium catalysis (ESC), met the requirement. The implementation of this innovation was first documented by Sharpless and co-workers in 1979 [10,11]. In the transformation, PhSeSePh was employed as the pre-catalyst, and underwent the secession of the Se-Se bond to generate an electrophilic species PhSeCl in the presence of *N*-chlorosuccinimide (NCS). Subsequent chloroselenenylation–deselenenylation process afforded allylic chloride and regenerated PhSeCl. After this seminal work, several oxidative systems such as PhSeSePh/persulfate [12,13,14,15,16,17,18,19], PhSeSePh/H_2_O_2_ [20,21,22,23,24,25], PhSeSePh/hypervalent iodide [26,27,28,29] and so on [30,31,32,33] have been developed in this realm [34,35,36]. Although considerable achievements have been made, limited transformations and rare effective asymmetric conversion necessitate a more fruitful oxidative system. Recently, a new oxidative system, the combination of electrophilic N-F reagents (Scheme 1) [37,38,39,40,41,42] and organoselenium compounds, has been applied in ESC process. This discovery promoted the development of impressive transformations and even asymmetric conversion over the past four years. The versatile reactivity, mild conditions, and excellent regio- and stereoselectivity of this catalytic system have attracted more and more attention of organic chemists. Consequently, this review is summarized to make a profile of recent development in ESC with electrophilic N-F reagents as the oxidants and reaction mechanisms are discussed concomitantly.

## 2. ESC with *N*-Fluorobenzenesulfonimide (NFSI) as the Oxidant

### 2.1. Allylic or Vinylic Imidation of Alkenes

Numerous biologically active compounds contain nitrogen atoms. Transition-metal catalyzed direct amination of alkenes is an efficient route for the synthesis of these compounds among the developed methods. The transformations generally went through the formation of C-N bond as a key step. However, there still existed some problems such as regio- and stereoselectivity, functional group tolerance and substrate scope. To address these issues, the development of efficient amination methods is desirable.

**Scheme 2:**
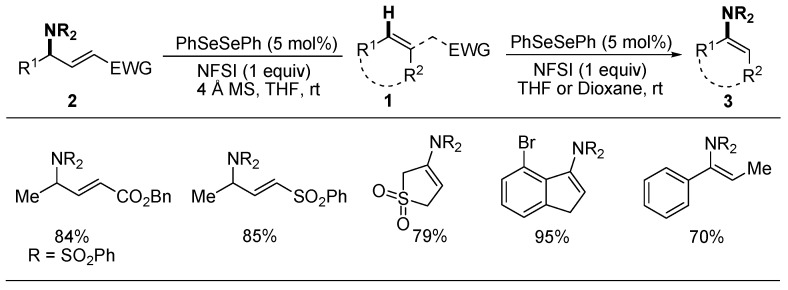
Organoselenium-catalyzed allylic and vinylic imidation of alkenes.

In 2013, Breder and co-workers disclosed an effective route to synthesize allylic and vinylic imidation products with olefins (**1**) by electrophilic selenium catalysis. *N*-Fluorobenzene-sulfonimide (NFSI) was utilized as the terminal oxidant and nitrogen source (Scheme 2) [43]. This work represents the first application of N-F reagents in organoselenium catalysis. As it is well known, halogenation products are formed in general when olefins react with N-X (X = Cl, Br, I) reagents in the presence of a catalytic amount of diphenyl diselenide (PhSeSePh) [10,11,44,45]. Surprisingly, the authors found that, when linear olefins tethered with an electron-withdrawing group reacted with NFSI in the presence of PhSeSePh, allylic imides (**2**) were generated smoothly instead of allylic fluorides [46]. By means of this method, γ^4^-amino acid derivatives, an important structure widely found in biological peptides, could be synthesized facilely. It should be point out that this protocol was compatible with cyclic olefins. However, vinylic imides (**3**) were obtained along with allylic imides in some cases [43].

**Scheme 3:**
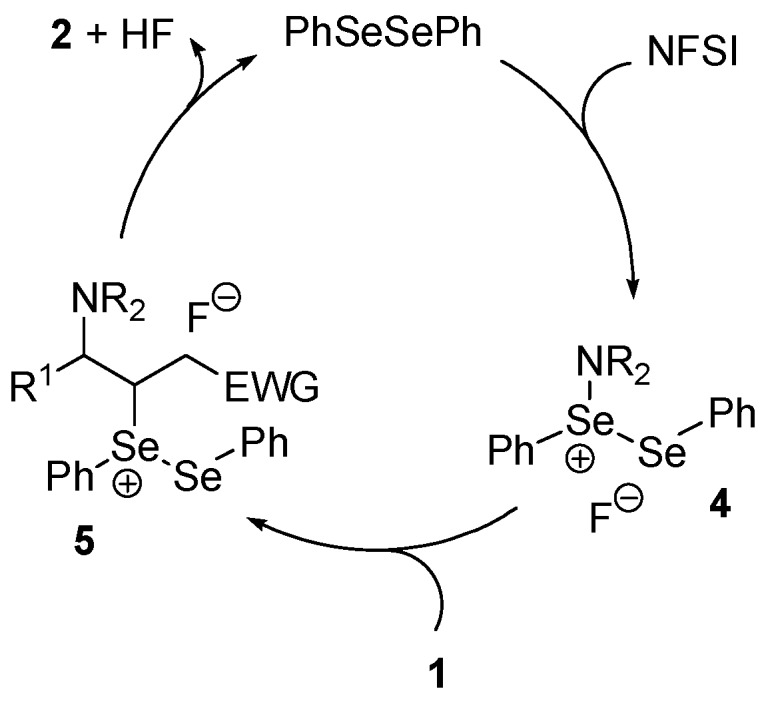
Proposed mechanism of allylic and vinylic imidation of alkenes.

No desired product was detected when the imidation proceeded without PhSeSePh under otherwise identical conditions. This ruled out the possibility of background reaction. Subsequent mechanistic studies revealed that the Se-Se bond was crucial for this transformation. Under the condition of PhSeBr as the catalyst, no imidated product was generated. Furthermore, it was found that both NFSI and PhSeSePh decomposed gradually in the NMR experimental studies when PhSeSePh was mixed with NFSI. However, NFSI did not further decompose after PhSeSePh was consumed completely. Thus, the authors speculated that the Se-Se bond did not break to form PhSeX (X = F or N(SO_2_Ph)_2_) species in the initial of the catalyzed reaction, and proposed a mechanism as follows (Scheme 3) [47]. The reaction is initiated by the formation of the ionic species **4**. Then, electrophilic attack of olefin affords intermediate **5**. Subsequent elimination gives rise to the desired product and the catalyst [43].

**Scheme 4:**
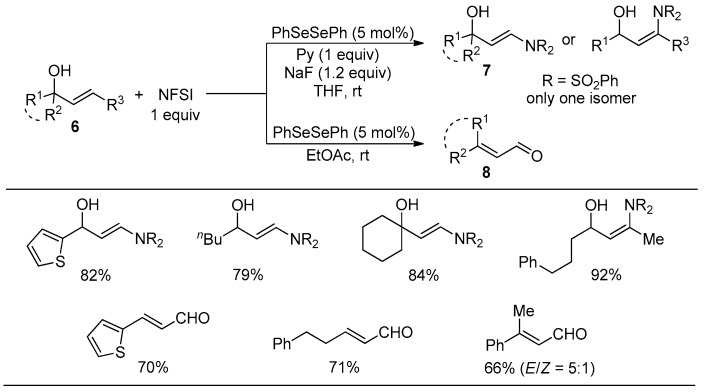
Organoselenium-catalyzed regio- and stereoselective imidation of terminal alkenes.

Successively, Zhao and co-workers reported an efficient route to synthesize 3-amino allylic alcohols through NFSI/PhSeSePh/base system (Scheme 4) [48]. 3-Amino allylic alcohols are valuable synthetic intermediates and generally obtained through multi-step synthesis. Inspired by the effect of neighboring group assistance, the authors proposed that 3-amino allylic alcohols could be synthesized conveniently via organoselenium catalyzed direct imidation of allylic alcohols. It was found that regio- and stereoselectivity of the reaction could be controlled by hydroxyl group on substrates [49,50,51,52,53]. When allylic alcohols (**6**) were treated with NFSI in the presence of base and a catalytic amount of PhSeSePh, only 3-amino allylic alcohols (**7**), an *anti*-Markovnikov product, were formed. Both terminal alkenes and 1,2-disubstituted alkenes also underwent the selective imidation to give the desired products under the similar conditions. Control experiments indicated that hydroxyl group was responsible for the excellent selectivity. When reaction of simple alkene without hydroxyl group was performed under the standard conditions, a mixture of allylic and vinylic imides was formed with erosion of stereoselectivity [48]. It is worth mentioning that, when allylic alcohols underwent the imidation without base, α,β-unsaturated aldehydes (**8**) were obtained due to the decomposition of 3-amino allylic alcohols in acidic conditions [54,55]. This is a new and practical method to synthesize α,β-unsaturated aldehyde derivatives with easily available allylic alcohols.

### 2.2. Synthesis of Isobenzofuranones and Indole Derivatives via Intramolecular C-H Functionalization

**Scheme 5:**
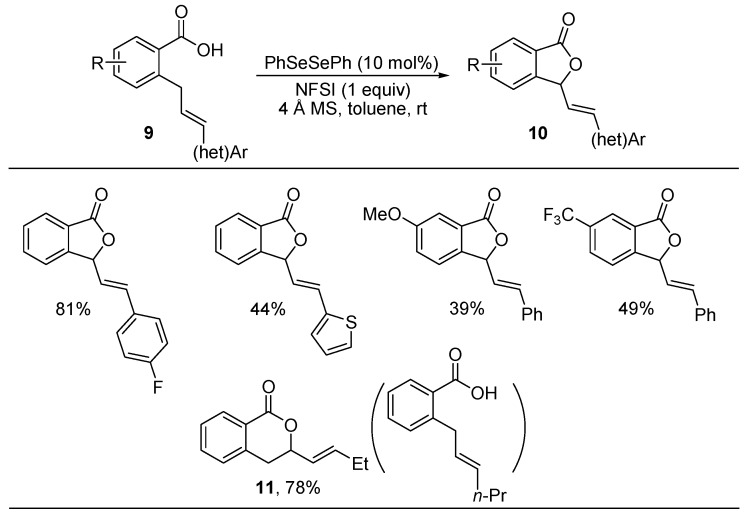
Organoselenium-catalyzed synthesis of isobenzofuranones.

By using NFSI as the oxidant in ESC process, several valuable heterocycles have been synthesized via oxidative cyclization. In 2015, Breder and co-workers developed a direct allylic acyloxylation protocol for the construction of isobenzofuranones [56]. When 2-cinnamylbenzoic acid analogues (**9**) were treated with NFSI in the presence of PhSeSePh, a variety of the corresponding isobenzofuranones (**10**) were generated in moderate to good yields (Scheme 5). However, the aryl groups connecting to the double bond were necessary for the successful implementation of this transformation. When aryl group was replaced by alkyl one, 6-*exo*-*trig* product **11** was afforded in 78% yield under the standard conditions [56].

**Scheme 6:**
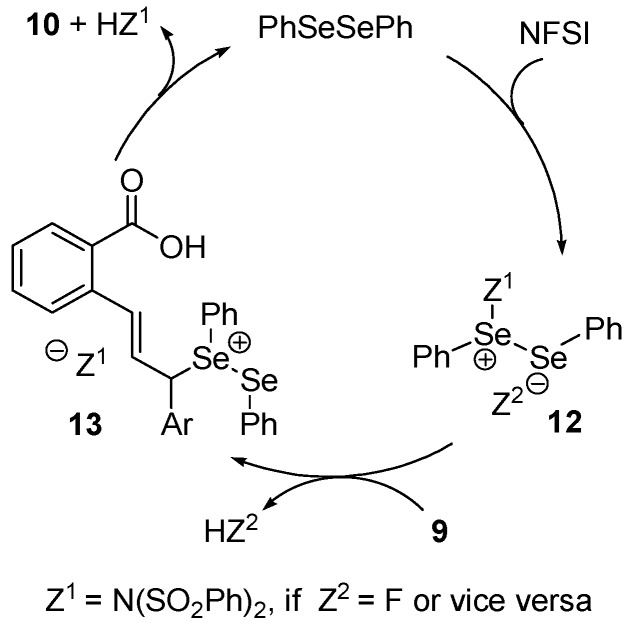
Proposed mechanism of direct allylic acyloxylation.

The role of diselenide in this transformation was appealing owing to the rarity of selenium catalyzed C(sp^3^)-H functionalization compared to hypervalent iodine catalysis [57,58]. According to a series of mechanistic studies, the authors rationalized that the reaction underwent allylic selenenylation-S_N_2’ displacement process (Scheme 6). Initially, diselenide is oxidized to electrophilic species **12**. Then, it reacts with olefinic acid **9** to form allylic selenium cationic **13**. Subsequent S_N_2’ displacement of selenium moiety releases the desired product **10** and regenerates the catalyst [56].

**Scheme 7:**
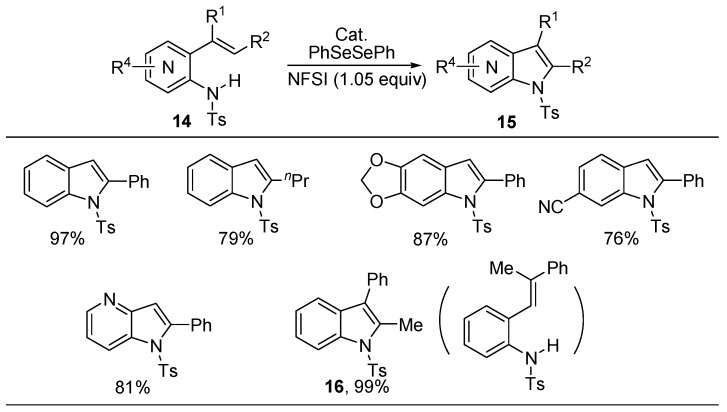
Organoselenium-catalyzed synthesis of indoles.

In further investigation of NFSI/PhSeSePh system in C-H functionalization, Breder and Zhao independently disclosed an effective route to synthesize indole derivatives via ESC process (Scheme 7) [59,60]. According to this procedure, 2-alkenylaniline (**14**) underwent the intramolecular C(sp^2^)-H amination in the presence of NFSI and PhSeSePh. Under the conditions, *N*-Ts-indole (**15**) derivative was obtained efficiently, but no intermolecular imidated product from NFSI was detected. In the case of tosyl, nosyl, and mesyl protected anilines, the corresponding products were afforded in moderate yields. However, no desired product was formed when Cbz, Ac, Boc and so on were utilized as the protecting groups. This finding indicated that the nucleophilicity of nitrogen atom is a key to the success of this transformation. This method is general, both 2-aryl and 2-alkyl indoles even azaindole derivatives could be synthesized in good yields. Furthermore, when trisubstituted alkene was treated with NFSI under the standard conditions, a 2,3-disubstituted *N*-Ts-indole **16** was formed in 99% yield via a 1,2-phenyl migration process [61,62,63]. In order to elucidate the mechanism, a cross experiment by using PhSeBr and tolylSeBr as the catalysts have been conducted by Breder and co-workers. The authors discovered that ArSeBr could catalyze the reaction and diselenides were generated after the full conversion of substrate. By analyzing ^77^Se NMR of the diselenide, two new signals were detected and putatively belonged to the mixed diselenide tolSeSePh. This compound was further confirmed by 2D ^77^Se, ^77^Se COSY experiments. According to these results, the Se-Se bond was possible to suffer cleavage/recombination during the catalytic reaction and oxyselenenylation–deselenenylation mechanism was reasonable for this cyclization [59].

## 3. ESC with Fluoropyridinium Salts as the Oxidants

### 3.1. Stereospecific Syn-Dichlorination of Alkenes

NFSI as the oxidant was efficiently utilized in ESC process. Versatile allylic or vinylic functionality compounds were synthesized by the oxidative system. Compared to NFSI, another type of electrophilic N-F reagents, fluoropyridium salts possess higher redox potential (Scheme 1) [42] and contain a weak nucleophile pyridine. Owing to the unique properties of these reagents, it might be promising to develop novel transformations with them and organoselenium compounds.

**Scheme 8:**
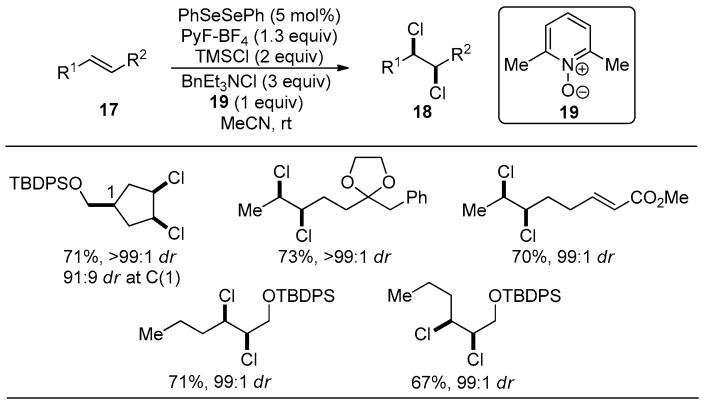
Organoselenium-catalyzed *syn*-dichlorination of alkenes.

In 2015, the first example of catalytic, stereoselective *syn*-dichlorination with simple olefins was disclosed by Denmark et al., and a novel PhSeSePh/PyF-BF_4_ system was employed in this transformation (Scheme 8) [64]. Owing to the inevitable *anti*-addition issue in traditional direct dichlorination of olefins via chloriranium ion, synthetic chemists were always plagued by the synthesis of *syn*-dichlorides. Inspired by the known feasibility of PhSeCl_3_ to afford *syn*-dichloride [65,66,67,68,69], the authors proposed a route of catalytic *syn*-dichlorination through ESC process. The critical point to accomplish this transformation was to identify an oxidant which satisfied some criteria: (1) it could oxidize Se (II) into Se (IV); (2) it could not react with substrates directly; (3) it could not oxidize Cl^−^ into Cl_2_ to avoid background reaction; (4) it could not release competitive nucleophiles compared to Cl^−^; and (5) it could promote the S_N_2 reaction of selenenylated intermediate rather than elimination [64].

**Scheme 9:**
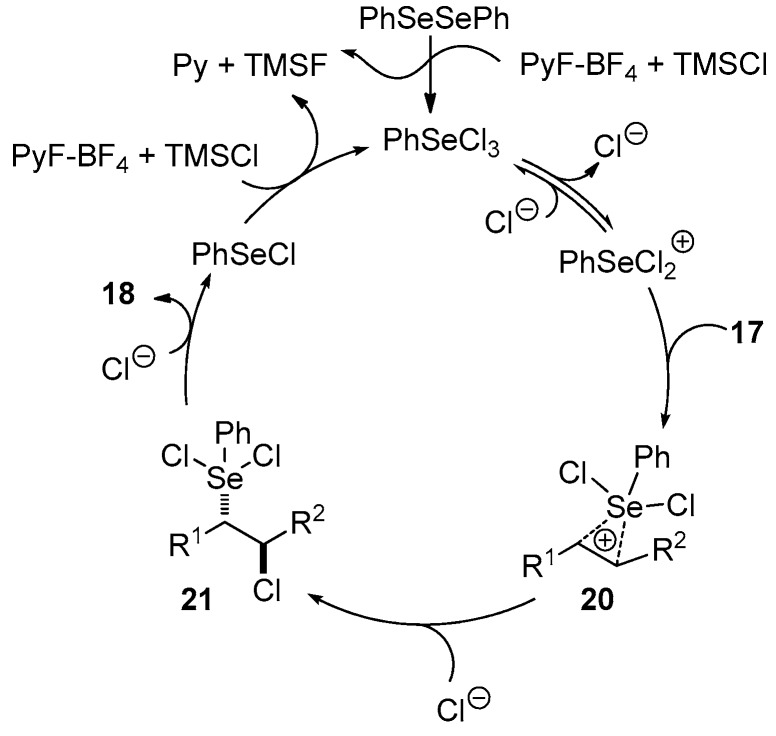
Proposed mechanism of *syn*-dichlorination of alkenes.

With these assumptions in mind, the authors speculated that N-F reagents might be a suitable oxidant. It was found that when simple alkenes (**17**) reacted with PyF-BF_4_ in the presence of BnEt_3_NCl, TMSCl and PhSeSePh, *syn*-dichlorides (**18**) were afforded stereoselectivitely in most cases. Additional 2,6-lutidine *N*-oxide (**19**) was able to accelerate the reaction. This transformation was compatible with a variety of functional groups including free hydroxyl group, *tert*-butyl-diphenylsilyl (TBDPS), acetal and so on. Control experiment revealed that ca. 35% *anti*-dichlorides were generated without PhSeSePh. It indicated that PyF-BF_4_ was capable of oxidizing Cl^−^ to Cl_2_ but the background reaction could be suppressed in the catalytic reaction. A possible mechanism of this catalytic *syn*-dichlorination is depicted as follows (Scheme 9). In the initial reaction, PhSeSePh is oxidized to PhSeCl_3_ species in the presence of PyF-BF_4_ and TMSCl. The PhSeCl_3_ might react with the alkene to generate seleniranium ion **20**. After the nucleophilic attack of Cl^−^, *anti*-stereospecific chloroselenenylated intermediate **21** is formed. Then, S_N_2 reaction of Se (IV) moiety by Cl^−^ releases the desired product **18** and PhSeCl [64].

### 3.2. Synthesis of Oxygen- and Nitrogen-Containing Heterocycles via Oxidative Cyclization

**Scheme 10:**
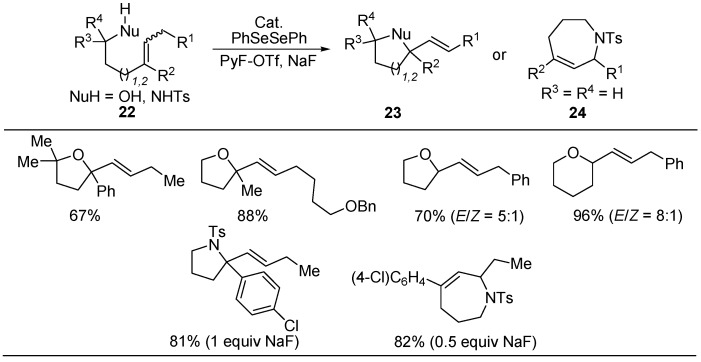
Organoselenium-catalyzed synthesis of oxygen- and nitrogen-containing heterocycles.

In 2016, Zhao and co-workers disclosed an organoselenium catalyzed oxidative cyclization to synthesize oxygen- and nitrogen-containing heterocycles [70]. The authors conceived that olefinic alcohols (**22**) were able to undergo *exo*-*trig* cyclization in the presence of PyF-OTf and PhSeSePh (Scheme 10). This protocol has been successfully applied into the formation of different α-alkenyl tetrahydrofurans or -pyrans (**23**) with excellent regio- and stereoselectivities. It is worth mentioning that, when NFSI or Selectfluor was employed as the oxidant, the reaction gave the product in lower yield with unidentified or fluorinated byproducts. This result emphasized an appropriate redox potential of oxidant was important for the transformation. Furthermore, the desired products were able to rearrange into seven-membered heterocycles (**24**) under the acidic conditions. In order to avoid the formation of byproducts, NaF was employed as the base to neutralize HF putatively generated in the reaction. It should be mentioned that olefinic amides also underwent the cyclization under the similar conditions. Both five- and seven-membered heterocycles were obtained in good yields by means of slight modification of bases. Some experiments have been conducted to elucidate the mechanism. It was found that a new signal was detected in ^77^Se NMR when PhSeSePh reacted with equimolar PyF-OTf after 4 h. When the mixture of PhSeSePh and PyF-OTf was employed to treat with olefinic alcohol, a selenenylated product was reasonably afforded in 61% NMR yield. Subsequent oxidation of this intermediate generated the desired product smoothly. Based on these results, the authors considered that PhSeSePh was initially oxidized to PhSeX (X = F or OTf) in the presence of PyF-OTf and the entire cyclization could undergo selenenylation–deselenenylation process [46,70].

### 3.3. Regioselective Pyridination of 1,3-Dienes

Selective functionalization of alkenes is a challenge in organic synthesis, especially for the regioselective C-H functionalization of 1,3-dienes due to the nature of the high reactivity of conjugated dienes. Many efforts have been devoted to this field. However, C-1 functionalization products were usually accessed by means of cross-coupling reactions [71,72,73,74,75,76,77]. However, there were rare documented transformations with respect to regioselective C-H functionalization at the other position of 1,3-dienes. Considering the excellent selectivity in selenenylation–deselenenylation process, electrophilic selenium catalysis could be an ideal strategy to overcome this issue.

**Scheme 11:**
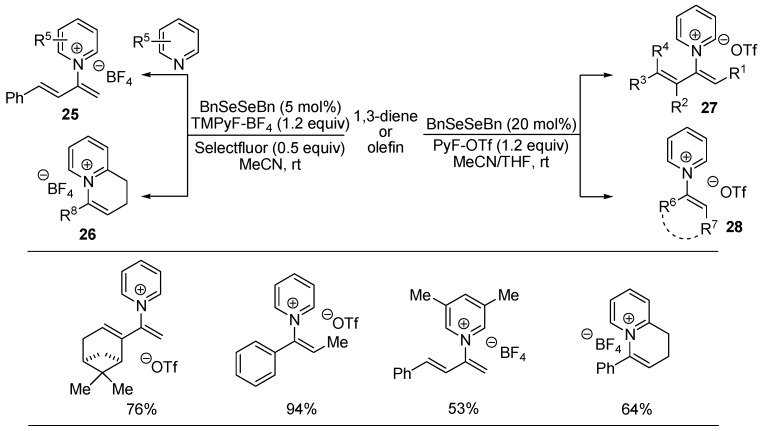
Organoselenium-catalyzed regioselective pyridination of 1,3-dienes.

Recently, Zhao and co-workers reported a regioselective C-H pyridination of olefins through BnSeSeBn/fluoropyridinium salts system [78]. In this transformation, fluoropyridinium salt served as pyridine source [79] and terminal oxidant (Scheme 11). The substrate scope of this transformation was broad. Different 1,3-dienes were suitable to the pyridination. Surprisingly, only C-2 pyridinated products (**27**) were formed by this method. Styrene derivatives were also compatible with the pyridination (see **28**). Moreover, exogenous and endogenous pyridine sources could be selectively installed on 1,3-dienes using co-oxidant TMPyF-BF_4_ and Selectfluor instead of PyF-BF_4_ (see **25** and **26**). It should be point out that when alkyl terminal alkenes were treated with fluoropyridinium salt under the standard conditions, a mixture of terminal pyridinated products was formed. This result emphasized the impact of conjugated aryl or vinyl group was crucial to achieve the remarkable selectivity. Further modification of pyridinium salts, such as Diels–Alder reaction, nucleophilic addition and aromatization, afforded valuable synthetic intermediates and demonstrated the potential applicability of this transformation [78].

**Scheme 12:**
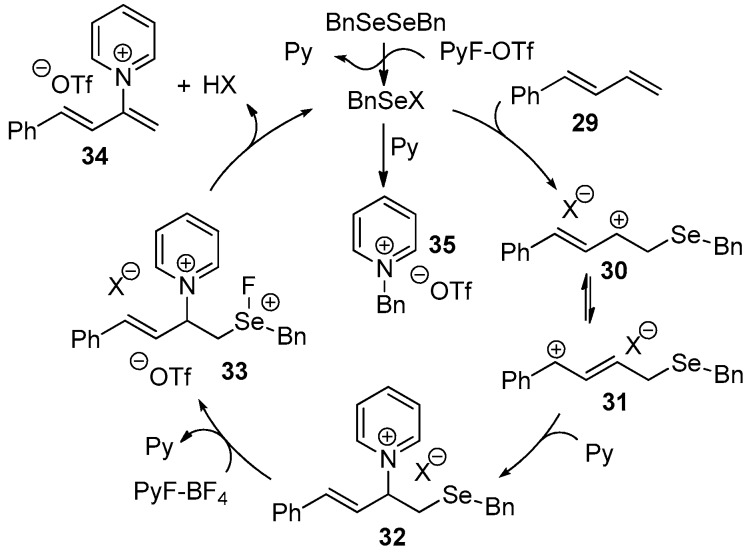
Proposed mechanism of regioselective pyridination of 1,3-dienes.

In order to make a profile of the selective pyridination [80,81,82], some mechanistic studies have been conducted. It was found that the selenenylated intermediate **32** along with *N*-benzyl-pyridinium salt **35** was formed when 1,3-diene **29** reacted with an equimolar mixture of BnSeSeBn and PyF-OTf. Further oxidation of **32** afforded the desired product **34** and byproduct **35**. These results proved that the selenenylation–deselenenylation process was rational to the pyridination. Therefore, the authors proposed that diselenide was initially oxidized into the real catalyst BnSeX. Then addition of 1,3-diene **29** forms intermediate **30** or **31**. Subsequently, pyridine selectively attacks to C-2 position owing to the more stability of intermediate **30**. After oxidation of selenenylated intermediate **32** and the following elimination, pyridinium salt **34** and RSeX species are produced (Scheme 12) [78].

## 4. Asymmetric Conversion

Asymmetric conversion plays an important role in modern synthesis owing to the realization of the different properties of enantiomer in biology. Over the past years, several ingenious organoselenium catalyzed enantioselective reactions have been developed, but most successful cases focused on the utilization of selenium in Lewis-base catalysis [83,84,85,86,87,88,89,90,91,92]. To date, efficient asymmetric conversion is still rare in electrophilic selenium catalysis although considerable efforts have been devoted in this field [93,94,95,96,97].

**Scheme 13:**
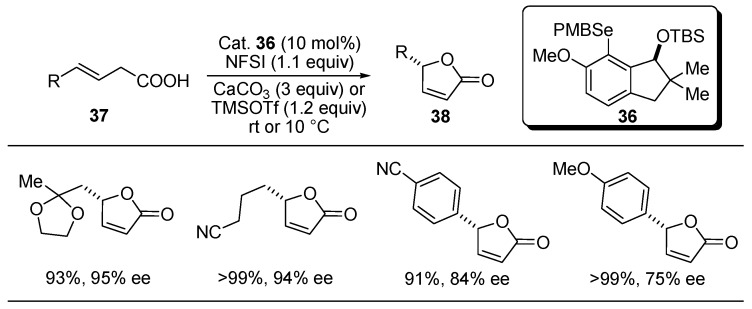
Organoselenium-catalyzed enantioselective synthesis of γ-lactones.

**Scheme 14:**
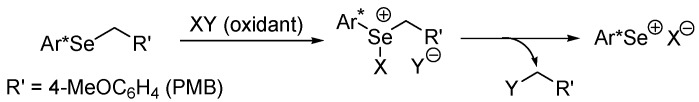
New route to generate electrophilic selenium catalyst with selenide.

In 2016, Maruoka and co-workers reported an enantioselective synthesis of γ-lactone through electrophilic selenium catalysis (Scheme 13) [98]. The desired products were obtained with excellent enantioselectivities because of the employment of a chiral selenide catalyst with the rigid indane scaffold (**36**). In electrophilic selenium catalysis, diselenides were generally utilized as the pre-catalysts. When the authors tried to synthesize indane-based diselenide, an inseparable mixture was formed. To solve this problem, they judiciously synthesized chiral indane-based selenide **36** bearing *p*-methoxy-benzyl group instead of the corresponding diselenide. It could also generate electrophilic selenium catalyst via an oxidative process (Scheme 14). To test the catalyst, β,γ-unsaturated carboxylic acid (**37**) was selected as the model substrate to undergo oxidative cyclization. Surprisingly, γ-lactones (**38**) were formed smoothly with high stereoselectivities in the presence of pre-catalyst **36** and NFSI. The authors mentioned that the *gem*-dimethyl group on catalyst was critical to the acquisition of high enantioselectivity. By this protocol, different kinds of alkyl β,γ-unsaturated carboxylic acids were converted into γ-lactones with excellent enantioselectivities ranging from 93% to 97%. Aryl substrates also underwent the lactonization with slightly erosion of enantioseletivities. It should be pointed out that the utilization of persulfate or hypervalent iodide as the oxidant led to the poor reactivity [98]. This result indicated the unique feature of N-F reagents in electrophilic selenium catalysis.

## 5. Conclusions

The application of N-F reagents in ESC process provides a powerful tool to functionalize the carbon–carbon double bond. Several elegant transformations have been developed by the catalytic system diselenide/N-F reagents. The common features of these reactions are easy to handle, mild conditions, excellent selectivity and good functional group tolerance. However, ESC process with N-F reagents is still in its infancy, especially in asymmetric conversion, which is a challenging issue. The current catalytic system is limited in the modification of alkenes. It has not been reported for its application in other unsaturated substrates such as allenes or alkynes. Furthermore, some details of this system are unclear and need to be further investigated.
